# Factors influencing decline of physical functional status among icu survivors: a prospective cohort study

**DOI:** 10.1186/2197-425X-3-S1-A361

**Published:** 2015-10-01

**Authors:** RPd Oliveira, R Rosa, A Ascoli, W Rutzen, L Madeira, F Ghizzoni, R Khummer, F Vargas, L Lago, C Dietrich, C Ceron, C Guterres, P Vesz, A Schaefer, M Falavigna, K Lima, C Robinson, R Ribeiro, J Maccari, C Teixeira

**Affiliations:** Moinhos de Vento Hospital, Porto Alegre, Brazil

## Introduction

Functional impairment has a direct impact on quality of life of ICU-survivors, because it limits autonomy and physical and mental abilities. So, these patients are more susceptible to chronic illness, increased long-term mortality.

## Objectives

This study sought to evaluate factors associated with decline of physical functional status in adult ICU survivors.

## Methods

A multicenter prospective cohort study was conducted with all consecutive adult patients admitted to mixed medical-surgical ICUs in Southern Brazil between May 2014 and December 2014. The decline of functional status in the period ranging from 3 months before ICU admission to 3 months after ICU discharge was evaluated through variations in Barthel index score. A stepwise multiple linear regression was performed to identify factors associated with decline of Barthel index score among ICU survivors.

## Results

In total, 99 patients (54% men) were evaluated during the study period. The mean age and APACHE-II score were 63.9 years (SD 16.6) and 13.3 points (SD 5.3), respectively. The mean ICU length of stay was 8.9 days (SD 10.0). The mean Barthel index score of the study population 3 months before ICU admission was 90.7 points (SD 16.6) while the mean Barthel index score 3 months after ICU discharge was 79.1 (SD 29.5). The Barthel index worsened in 49.5%, improved in 10.1% and did not change in 40.4% of patients. Of the 27 patients who were active workers, only 13 (48.1%) returned to work. According to multiple linear regression analysis, age (β=-0.30, *p* = 0.02) and need of mechanical ventilation during ICU stay (β=-13.40, *p* = 0.02) were independently associated with decline of physical functional status.

## Conclusions

Physical functional status 3-months after ICU-discharge may be significantly poorer in elderly and mechanical ventilation dependent patients.
